# Associations of acute conflict with equity in maternal healthcare: an uncontrolled before-and-after analysis of Egypt demographic and health survey data

**DOI:** 10.1186/s12939-018-0845-6

**Published:** 2018-08-29

**Authors:** Saji Saraswathy Gopalan, Richard Silverwood, Natasha Howard

**Affiliations:** 1 Department of Global Health and Development, London School of Hygiene and Tropical Medicine, London, UK; 2 Department of Medical Statistics, London School of Hygiene and Tropical Medicine, London, UK

**Keywords:** Egypt, Maternal health, Conflict, Equity, DHS

## Abstract

**Background:**

Equity of usage of maternal services during conflict is considered key to reducing maternal health risks globally. However, evidence showing how conflict affects maternal care use among different population groups is minimal. This study examined how the Egyptian acute conflict of 2011–2012 affected maternal care use among different socioeconomic, demographic, and geographic groups.

**Methods:**

An ‘uncontrolled before-and-after’ study design was used to perform multi-level modelling regression analysis on 2014 Egypt Demographic and Health Survey data. The pre-conflict sample included 2569 births occurring from January 2009 to January 2011 and the peri-conflict sample included 4641 births from February 2011 to December 2012.

**Results:**

Interaction analysis indicated that the effect of conflict on some aspects of maternal care differed by mother’s age, residential status, employment, education level and household wealth. In the stratum-specific analysis, increased odds of skilled delivery during conflict was relatively greater among women who were rural (odds ratio [OR] 1.02; 95%CI 1.02–1.03), educated to primary level (OR 1.04; 95%CI 1.01–1.07), employed (OR 1.04; 95%CI 1.01–1.07), less poor (OR 1.03; 95%CI 1.02–1.05) or middle-income (OR 1.02; 95%CI 1.01–1.04), than pre-conflict. Similarly, increased odds of physician-assisted delivery during conflict was relatively greater for women who were rural (OR 1.03; 95%CI 1.02–1.04), educated to primary level (OR 1.05; 95% CI 1.01–1.10), employed (OR 1.07; 95%CI 1.02–1.11), or from less poor/middle-income (OR 1.03; 95%CI 1.01–1.05 each), and richest quintiles (OR 1.02; 95%CI 1.00–1.03). Decreased odds of postnatal care during conflict was relatively greater among women aged 25–29 (OR 0.92; 95%CI 0.88–0.96) compared to older women.

**Conclusions:**

The association between acute conflict and maternal services usage indicated some vertical equity, as equity patterns during conflict differed from recent trends in Egypt. The association between conflict and maternal care usage among potentially marginalised groups was minimal and not notably inequitable. Specific strategies should be included in maternal health policies to mitigate the unpredictable effect of conflict on maternal care equity. Further research is needed to determine how conflict affects out-of-pocket expenditures and quality-of-care among different socioeconomic groups.

## Background

Equitable access to and usage of appropriate and good-quality maternal care is a key challenge in addressing maternal and neonatal health in low and middle-income countries (LMICs), including conflict-affected settings [1]. Improving health equity includes removing unfair and avoidable differences in healthcare access and usage among populations, e.g. those based on socioeconomic, demographic, or geographical status [2–4]. Nearly 60% of global maternal deaths occur in conflict-affected LMICs [1, 5], with a maternal mortality ratio of 417 per 100,000 live births in 2014 [1]. In the same year, skilled birth attendance (SBA) was 85% higher among the richest women in LMICs, while the chances of four or more antenatal care visits were 25% higher among highest educated women [6]. A study conducted in 19 conflict-affected LMICs reported that mean access to SBA among the lowest socioeconomic quintile was 12.2% compared to 81.2% among the richest quintile [2]. The United Nations’ (UN) Sustainable Development Goal agenda recommends reducing inequities in maternal care access in conflict-affected LMICs to improve maternal health [5]. However, evidence is limited on maternal care-seeking patterns and how conflict affects equity in access to or usage of maternal healthcare in LMICs.

Health equity is a complex concept to define and measure, particularly in conflict-affected settings [2, 7, 8]. It can be categorised as horizontal or vertical [2, 7, 8]. Horizontal equity determines that women with the same health needs are treated equally, irrespective of morally irrelevant factors (e.g. age, ethnicity, income, autonomy, ability to benefit etc.) [2, 7, 8]. For example, women from the same socioeconomic background are treated equally by caregivers without any discrimination [2, 7, 8]. Vertical equity determines that women with differing health needs are prioritized differently, according to morally irrelevant factors (e.g. age, autonomy, ability to benefit, income etc.) [2, 7, 8]. These differing health needs are based on their current status of social determinants. For example, poor women might receive more government support than rich women due to relatively higher economic vulnerability [7, 8].

The Egyptian healthcare system was identified as one with increasing inequities in the social determinants of maternal care and services availability, access, and usage [9]. It was thus relevant to assess how women with unequal endowments of social determinants (e.g. wealth) used maternal services during the acute 2011–2012 conflict. Literature and policy evidence, from LMICs and resource-constrained settings, indicates that vertical equity should be addressed prior to horizontal equity [10, 11]. Unless disadvantaged groups are given differential treatment, prevailing gaps in health status will not be reduced substantially [10, 11]. Thus, this study focused where possible on vertical equity in usage of maternal care services during the acute Egyptian conflict.

Evidence from conflict-affected countries shows that conflicts adversely affect population health and maternal care by restricting timely availability and access to quality care [12–14]. Addressing equity in maternal care is challenging in conflict-affected settings, particularly during acute conflict [4, 15–17]. Conflict and violence can reduce health system capacity for equitable health service delivery [4, 16]. Social determinants of health may function less predictably during conflict [3], e.g. violence may prevent facility access even if financial barriers are removed [18, 19]. Lack of reliable data on maternal care use and equity patterns during conflict limits health system preparedness to support women during conflicts [15, 17]. In this context, this study tested the association between the acute 2011–2012 Egyptian conflict and maternal care usage among different socioeconomic, demographic, and geographic groups.

Egypt was chosen as a case study because the 2014 DHS provided one of the only robust datasets that enabled a before-and-after comparison of the association of conflict with equity of maternal care usage. Evidence was limited on the 2011–2012 Egyptian conflict’s effect on maternal care in general and equity dimensions in particular [20]. Egypt is considered at risk of further conflict, yet despite health system reforms in the last decade, policy attention to building resilience to conflict has been limited [21, 22]. A better understanding of the socioeconomic determinants of maternal care could help inform policy in addressing equity during future conflicts [21]. Additionally, maternal care in Egypt has become increasingly inequitable in the past decade [22, 23]. For example, a recent study reported that a one unit increase in the mean socio-cultural resourcefulness score was associated with 1.55 higher odds of using any antenatal care (ANC) and 1.31 higher odds of institutional delivery [9]. Rural areas have a relatively lower percentage (20%) of the total health centres nationally [23–25]. How predictable social determinants of maternal care are during acute conflict and whether maternal care use changes differently during conflict among different socioeconomic groups are also not that known in the Egyptian context.

This study aimed to assess the association between the 2011–2012 Egyptian conflict and usage of maternal services among women in different socioeconomic, demographic and geographic groups in order to examine vertical equity. Objectives were to compare usage of antenatal, delivery, and postnatal services before and during the conflict, by: (i) maternal age; (ii) residence; (iii) education; (iv) employment; and (v) household wealth.

## Methods

### Study setting

Egypt is a lower-middle-income country in northern Africa with a Gross National Income per capita of US$ 5654 in 2012 [10]. The pluralistic health system is more poorly resourced (i.e. staff, funding, supplies, infrastructure) in rural areas compared to urban areas, an indication of geographic inequity [11]. Under-five mortality during 2008–2013 averaged 27 deaths per 1000 births [11]. The maternal mortality ratio was 33 per 100,000 live births in 2014 [26]. Poor quality-of-care and delays in seeking care were identified as major causes of maternal death in Egypt [27, 28].

**Fig. 1:**
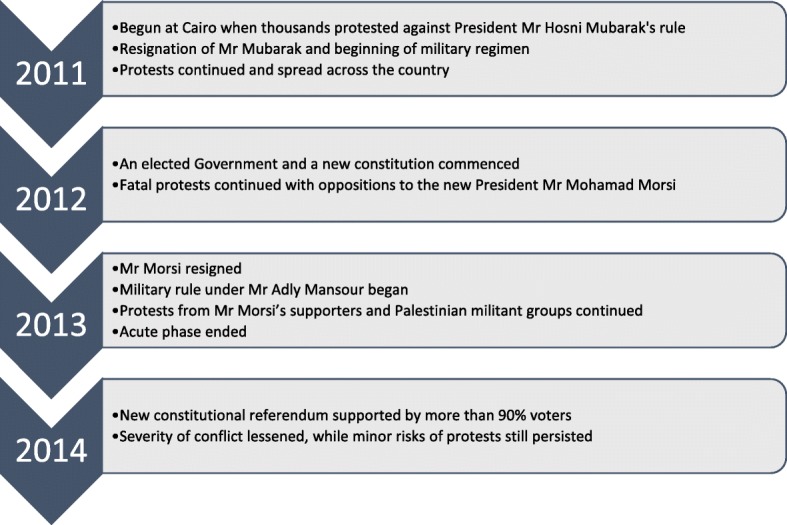
Chronology of events related to Egyptian conflict 2011. Adapted from Abdou DS [30] and Maher S [29]

The Egyptian revolution started in January 2011, when thousands of civilians protested against the government, eventually leading to the resignation of long-time president Mr. Hosni Mubarak [29, 30]. While the acute phase ended early in 2013 [29], Egypt is not considered fully free from the threat of civil unrest. Despite an elected government taking office in 2012, political protests continued [30]. Several socio-political and economic reasons behind the revolution have not been addressed, including rising poverty, perceived government autocracy, and neglect of social welfare [29]. Although evidence is still emerging, conflict is considered to have adversely affected Egypt’s economic and human development indicators [30]. Figure 1 provides a chronology of events related to the conflict.

### Study design

A quasi-experimental ‘uncontrolled before-and-after’ design was selected, using multi-level modelling (MLM) regression of 2014 Egypt Demographic and Household Survey (EDHS) data, to compare levels of maternal care usage before and during the acute 2011–2013 Egyptian conflict across socioeconomic groups. The study adopted a working definition of ‘conflict-affected’ from relevant literature as a setting in which routine socio-political, economic and/or civil life are disrupted due to armed political conflict [16, 31–34]. The ‘pre-conflict’ sample included births from January 2009 to January 2011 while the ‘peri-conflict’ sample included births from February 2011 to December 2012. Based on media reports describing the end of the acute phase of the conflict in early 2013, births from January 2013 onwards were excluded [29]. Analysis thus included 7210 births from 7118 eligible women in 1679 clusters (i.e. 2569 pre-conflict births and 4641 peri-conflict births).

### Sampling and data collection

Data were drawn from the 2014 EDHS, which provided a nationally-representative sample (excluding North and South Sinai governorates) [21]. The 2014 EDHS used multi-stage sampling [21]. First,884 primary sampling units (PSUs) were selected. Second, depending on PSU size, systematic sampling yielded 1–3 parts per PSU (1000 households each). Third, each part was divided into equally-sized segments (200 households each), two to three of which were selected randomly from each PSU. A total of 1838 segments (clusters) were selected from 884 PSUs. A household listing was undertaken in each segment. An average of 15 households was selected from each segment using systematic random sampling. Thus, a total of 29,471 households were included. Eligible participants were ever-married women aged 15–49 and present in selected households the night prior to interview. A total of 21,903 women were eligible for the survey. From each eligible woman, details were gathered on childbirths in the five years preceding the date of survey. The 2014 EDHS response rate was 98.4%.

Data were collected in April–June 2014 [21]. Maternal data, collected by local recently-graduated enumerators in Arabic, included place of care-seeking, type of facility, provider and frequency of attendance, timeliness, and contents of services received. All questions were pre-tested and revised based on comments from interviewers, pre-testing, and tabulations of pre-test results. As part of quality control, enumeration teams were closely supervised throughout fieldwork and field editors conducted routine re-interviews using a shortened questionnaire.

### Outcome and explanatory variables

Outcome variables were usage of seven binary maternal care components, selected based on data reliability and relevance to improve maternal and neonatal health in a LMIC setting based on empirical evidence [35, 36]:

- antenatal care: (i) 4+ ANC visits completed, (ii) ANC from government provider, (iii) ANC from a doctor.
- delivery care: (iv) delivered with a skilled attendant, (v) delivered with a doctor, (vi) delivered in a public facility.
- postnatal care (PNC): (vii) any PNC received.

Selection of outcome measures was based on WHO’s list of essential maternal care services for LMICs and Egypt’s maternal care clinical guidelines [36, 37]. These maternal care indicators consider social and health determinants of maternal care including service delivery in LMICs [35, 36].

Explanatory variables were five maternal characteristics: (i) age, (ii) urban/rural residence, (iii) education, (iv) employment, and (v) household wealth status. Household wealth was calculated in the 2014 EDHS dataset in quintiles from poorest to wealthiest. EDHS calculations used a step-wise approach with key household economic characteristics and assets weighted using principal component analysis [21] .

### Analysis

Data were analysed, using Stata software version 13, both descriptively and by estimating effects through multilevel modelling. Descriptive analyses summarised key explanatory variables by period (pre-conflict, peri-conflict) with frequencies for categorical variables. Multilevel regression models were developed to account for data clustering and allow for the dependency of observations within clusters [38–40]. EDHS data are hierarchical, i.e. births are nested within households, households within clusters, clusters within PSUs, and PSUs within Governorates [21], and conventional linear regression models that do not account for multiple levels would underestimate the standard errors of effect sizes with a higher chance of Type I error [38–40]. In hierarchical data, multi-level modelling accounts for dependency of observations and covariates at multiple levels, without assigning them to one or limited levels only [38].

The multilevel model accounted for sample hierarchy at four levels, with a random intercept at each, excluding level 1: births (level 1), household nested within clusters (level 2), cluster nested within PSUs (level 3), and PSU nested within governorates (level 4). MLM included fixed effects of individual factors [38]. Logistic regressions were performed, adjusting for maternal age, residence, education, employment, and household wealth. The sampling weights, applied by EDHS statisticians for national representativeness, were accounted for in multilevel regressions. Sampling weights were rescaled, since including raw weights without scaling in MLM could lead to biased parameters and standard errors [38], so that the new weights would sum to the effective cluster size [38].

Multilevel models were applied by socioeconomic strata as per the following model specifications:

$$ \log \left(\frac{{\mathrm{Y}}_{\mathrm{ijkl}}}{1-{\mathrm{Y}}_{\mathrm{ijkl}}}\right)=\propto +{\upbeta}_1{\mathrm{X}}_{1\mathrm{ijkl}}+\dots +{\upbeta}_n{\mathrm{X}}_{\mathrm{nijkl}}+{\upgamma \mathrm{T}}_{\mathrm{ijkl}}+{\uptheta}_{\mathrm{l}}+{\mu}_{kl}+{\upeta}_{jkl} $$

where Y_ijkl_ is the outcome for birth i (level 1), within cluster j (level 2), within PSU k (level 3), within governorate l (level 4). α is a constant and X_1ijkl ……._ X_nijkl_ are the explanatory variables, with β_1 ……_ βn as their coefficients. T_ijkl_ is a binary variable that is 1 for the ‘peri-conflict’ sample and 0 otherwise, with γ as its coefficient. θ_l_, μ_kl_ and η_jkl_ are the error terms at governorate, PSU and cluster levels respectively.

Next, interactions between conflict and each explanatory variable were estimated in turn as per the following model specification:

$$ \log \left(\frac{{\mathrm{Y}}_{\mathrm{ijkl}}}{1-{\mathrm{Y}}_{\mathrm{ijkl}}}\right)=\propto +{\upbeta}_1{\mathrm{X}}_{1\mathrm{ijkl}}+\dots +{\upbeta}_n{\mathrm{X}}_{\mathrm{nijkl}}+{\upgamma \mathrm{T}}_{\mathrm{ijkl}}+\uprho {\left(\mathrm{T}.\mathrm{X}\right)}_{\mathrm{ijkl}}+{\uptheta}_{\mathrm{l}}+{\mu}_{kl}+{\upeta}_{jkl} $$

where Y_ijkl_ is the outcome for birth i (level 1), within cluster j (level 2), within PSU k (level 3), within governorate l (level 4). α is a constant and X_1ijkl ……._ X_nijkl_ are the explanatory variables, with β_1 ……_ βn as their coefficients. T_ijkl_ is a binary variable that is 1 for the ‘peri-conflict’ sample and 0 otherwise, with γ as its coefficient. T.X is the interaction term between conflict and the given explanatory variable, with ρ as its coefficient. θ_l_, μ_kl_ and η_jkl_ are the error terms at governorate, PSU, and cluster levels respectively.

Finally, a joint test of the interaction terms was performed using a Wald test to assess whether the effect of conflict on each outcome variable varied significantly between strata for a given explanatory variable.

## Results

### Sample characteristics

Table 1 shows descriptive analysis of key socio-demographic variables. The pre-conflict sample included 2569 births and the peri-conflict sample included 4641 births. The DHS report indicated that this near doubling of births in the peri-conflict period compared to the pre-conflict period was due to an unusual national doubling of births from 2011 to early 2013 [21]. For example, the total number of stillbirths to women aged 15–49 was 29,349 during 2012–2014 and only 8109 during 2009–2010 [21]. In the pre-conflict period, 66% of births were to women aged 30+ years, peri-conflict this was 41%. There was a marked shift to younger maternal age between pre-conflict and peri-conflict periods and this trend of early childbirth among women in Egypt and the region has been widely discussed [21, 41]. The majority of births were to women with secondary or higher education (58% pre-conflict, 62% peri-conflict) and from rural settings (65% pre-conflict, 69% peri-conflict). Above half of women gave birth to a male baby (55% pre-conflict, 53% peri-conflict) in their last birth preceding the survey. The largest wealth quintile was the middle (24% pre-conflict, 25% peri-conflict). A majority of births were to non-working women (85% pre-conflict, 87% peri-conflict). Slightly above half of women already had 2–3 children (60% pre-conflict, 54% peri-conflict). Nearly all women (97%) were Muslim, while the rest were Christian.

**Table 1:** Sample characteristics

Characteristics	Pre-conflict *N* = 2569		Peri-conflict *N* = 4641	
	n	%	n	%
Age group				
< 25	157	6.1	1040	22.4
25–29	709	27.6	1713	36.9
30–34	853	33.2	1091	23.5
> 35	850	33.1	798	17.2
Education				
No education	550	21.4	840	18.1
Primary	524	20.4	937	20.2
Secondary and above	1495	58.2	2863	61.7
Residence				
Urban	897	34.9	1429	30.8
Rural	1672	65.1	3212	69.2
Gender of child				
Male	1413	55	2455	52.9
Female	1156	45	2186	47.1
Wealth index				
Poorest	465	18.1	784	16.9
Poorer	504	19.6	928	20
Middle	611	23.8	1165	25.1
Richer	516	20.1	993	21.4
Richest	473	18.4	770	16.6
Currently working				
No	2181	84.9	4024	86.7
Yes	388	15.1	617	13.3
Birth order				
1	252	9.8	1109	23.9
2–3	1544	60.1	2506	54
4–5	622	24.2	863	18.6
6 and above	154	6	162	3.5
Religion				
Muslim	2479	96.5	4488	96.7
Christian	90	3.5	158	3.4

### Association between conflict and maternal services usage by maternal age

Table 2 provides the stratified analysis of adjusted associations of age-group with maternal outcomes in the peri-conflict period compared to the pre-conflict period. The joint interaction test suggested that the effect of conflict on maternal care use differed by age group for several outcomes: receiving ANC from a government provider (*p* = 0.003), receiving ANC from a doctor (*p* < 0.001), delivering in public institutions (*p* = 0.01) and receiving any PNC (*p* = 0.02). In the stratified analysis, the reduction in the odds of receiving ANC from a government provider during conflict was relatively greater for younger women (e.g. age < 25: OR 0.91; 95% CI 0.83–0.99) than for older women (e.g. age > 35: OR 0.98; 95% CI 0.95–1.02); the increase in the odds of receiving ANC from a doctor during conflict was relatively greater for younger women (e.g. age < 25: OR 1.09; 95% CI 0.95–1.25) than for older women (e.g. age > 35: OR 0.99; 95% CI 0.98–1.01); the reduction in the odds of delivering in a public institution during conflict was relatively greater for younger women (e.g. age < 25: OR 0.93; 95% CI 0.87–0.99) than for older women (e.g. age > 35: OR 0.99; 95% CI 0.96–1.02). The association with receiving any PNC was more complicated, with women aged 25–29 having the greatest reduction in odds during conflict (OR 0.92; 95% CI 0.88–0.96) and women aged above 35 having the greatest increase (OR 1.05; 95% CI 0.99–1.11).

**Table 2:** Multilevel modelling estimates of the association between conflict and maternal care usage by maternal age

Period	< 25 years (*n* = 457)	25–29 years (*n* = 1749)	30–34 years (*n* = 1704)	> 35 (*n* = 1532)	Wald *p* value^#^
	OR^a^ (95%CI)	OR^a^ (95%CI)	OR^a^ (95%CI)	OR^a^ (95%CI)	
4+ ANC visits					
Pre	1.00 (Reference)	1.00 (Reference)	1.00 (Reference)	1.00 (Reference)	0.48
Peri	1.00 (0.92–1.09)	1.00 (0.94–1.06)	1.02 (0.99–1.05)	1.00 (0.95–1.05)	
ANC from a government provider					
Pre	1.00 (Reference)	1.00 (Reference)	1.00 (Reference)	1.00 (Reference)	0.003
Peri	0.91*(0.83–0.99)	0.96 (0.93–1.00)	1.01* (1.00–1.02)	0.98 (0.95–1.02)	
ANC from a doctor					
Pre	1.00 (Reference)	1.00 (Reference)	1.00 (Reference)	1.00 (Reference)	< 0.001
Peri	1.09* (0.95–1.25)	1.04 (0.96–1.12)	0.97 (0.94–1.01)	0.99 (0.98–1.01)	
Delivery by skilled provider					
Pre	1.00 (Reference)	1.00 (Reference)	1.00 (Reference)	1.00 (Reference)	0.70
Peri	0.99 (0.92–1.07)	1.01 (0.99–1.03)	1.01 (0.99–1.03)	1.02 (0.99–1.04)	
Delivery by doctor					
Pre	1.00 (Reference)	1.00 (Reference)	1.00 (Reference)	1.00 (Reference)	0.94
Peri	0.96 (0.87–1.06)	1.02 (0.99–1.06)	1.02 (0.98–1.04)	1.03*(1.00–1.06)	
Delivery in public institution					
Pre	1.00 (Reference)	1.00 (Reference)	1.00 (Reference)	1.00 (Reference)	0.01
Peri	0.93*(0.87–0.99)	0.99 (0.95–1.03)	1 (0.96–1.04)	0.99 (0.96–1.02)	
Any PNC					
Pre	1.00 (Reference)	1.00 (Reference)	1.00 (Reference)	1.00 (Reference)	0.02
Peri	0.98 (0.87–1.11)	0.92***(0.88–0.96)	1.03 (0.99–1.08)	1.05 (0.99–1.11)	

^a^ Multilevel modelling estimates adjusted for education, residence, child gender, household wealth status, currently working status and birth order; * < 0.05; ** < 0.01; *** < 0.001; sample size is pre-conflict 2569 and peri-conflict 4641;# joint interaction term explaining the effect of conflict on outcome variable between strata

### Association between conflict and maternal services usage by maternal residence

In Table 3, the interaction test showed that the effect of conflict differed by maternal residence for receiving ANC from a government provider (*p* = 0.01), SBA (*p* < 0.001), physician-assisted delivery (*p* < 0.001), and delivery in public institutions (*p* = 0.03). In the stratified analysis, the reduction in the odds of receiving ANC from a government provider during conflict was relatively greater for rural women (OR 0.99; 95% CI 0.98–0.99) than for urban women (OR 0.99; 95% CI 0.96–1.01); the increase in odds of receiving SBA during conflict was relatively greater for rural women (OR 1.02; 95% CI 1.02–1.03) than for urban women (OR 1.00; 95% CI 0.99–1.01); the increase in odds of receiving physician-assisted delivery during conflict was relatively greater for rural women (OR 1.03; 95% CI 1.02–1.04) than for urban women (OR 1.01; 95% CI 0.98–1.04); and the reduction in odds of public institutional delivery during conflict was relatively greater for urban women (OR 0.97; 95% CI 0.95–0.99) than for rural women (OR 1.00; 95% CI 0.98–1.01).

**Table 3:** Multilevel modelling estimates of the association between conflict and maternal care usage by maternal residence

Period	Rural (*n* = 3188)	Urban (*n* = 2254)	Wald *p* value^#^
	OR^a^ (95% CI)	OR^a^ (95% CI)	
4+ ANC visits			
Pre	1.00 (Reference)	1.00 (Reference)	0.36
Peri	1.01 (1.00–1.02)	1.01 (0.99–1.03)	
ANC received from a government provider			
Pre	1.00 (Reference)	1.00 (Reference)	0.01
Peri	0.99***(0.98–0.99)	0.99 (0.96–1.01)	
ANC received from a doctor			
Pre	1.00 (Reference)	1.00 (Reference)	0.83
Peri	1.00 (0.96–1.05)	1.01 (0.99–1.03)	
Delivery by skilled provider			
Pre	1.00 (Reference)	1.00 (Reference)	< 0.001
Peri	1.02*** (1.02–1.03)	1.00 (0.99–1.01)	
Delivery by doctor			
Pre	1.00 (Reference)	1.00 (Reference)	< 0.001
Peri	1.03*** (1.02–1.04)	1.01 (0.98–1.04)	
Delivery in a public institution			
Pre	1.00 (Reference)	1.00 (Reference)	0.03
Peri	1.00 (0.98–1.01)	0.97* (0.95–0.99)	
Any PNC			
Pre	1.00 (Reference)	1.00 (Reference)	0.53
Peri	1.01 (0.97–1.04)	1.01 (0.99–1.03)	

^a^ Multilevel modelling estimates adjusted for education, residence, child gender, household wealth status, currently working status and birth order; * < 0.05; ** < 0.01; *** < 0.001; sample size is pre-conflict 2569 and peri-conflict 4641;# joint interaction term explaining the effect of conflict on outcome variable between strata

### Association between conflict and maternal services usage by maternal education

In Table 4, the joint interaction test showed that the effect of conflict differed by maternal education level for SBA (*p* < 0.001), physician-assisted delivery (*p* < 0.001), and delivery in public institutions (*p* = 0.01). In the stratified analysis, the increase in the odds of receiving SBA during conflict was relatively greater for women educated to primary level (OR 1.04; 95% CI 1.01–1.07) than for women with no education (OR 1.00; 95% CI 0.96–1.05) or women educated to secondary level (OR 1.00; 95% CI 0.99–1.01); the increase in the odds of doctor-assisted deliveries during conflict was relatively higher for women educated to primary level (OR 1.05; 95% CI 1.01–1.10) than for women with no education (OR 1.00; 95% CI 0.97–1.05) or women educated to secondary level (OR 1.01; 95% CI 1.00–1.03); and the reduction in the odds of delivery in a public institution was relatively greater for women educated to secondary level (OR 0.97; 95% CI 0.96–0.99) than for women with no education (OR 1.01; 95% CI 0.95–1.07) or women educated to primary level (OR 1.02; 95% CI 0.99–1.05).

**Table 4:** Multilevel modelling estimates of the association between conflict and maternal care usage by maternal education level

Period	No education (*n* = 1109)	Primary (*n* = 1123)	Secondary (*n* = 3210)	Wald *p* value^#^
	OR^a^ (95% CI)	OR^a^ (95% CI)	OR^a^ (95% CI)	
4+ ANC visits				
Pre	1.00 (Reference)	1.00 (Reference)	1.00 (Reference)	0.07
Peri	0.99 (0.98–1.00)	1.00 (0.97–1.03)	1.02 (1.00–1.04)	
ANC received from a government provider				
Pre	1.00 (Reference)	1.00 (Reference)	1.00 (Reference)	0.42
Peri	1.00 (0.94–1.05)	0.98 (0.92–1.05)	0.98 (0.96–1.00)	
ANC received from a doctor				
Pre	1.00 (Reference)	1.00 (Reference)	1.00 (Reference)	0.38
Peri	1.01 (0.95–1.07)	0.96 (0.84–1.10)	1.01 (0.98–1.04)	
Delivery by skilled provider				
Pre	1.00 (Reference)	1.00 (Reference)	1.00 (Reference)	< 0.001
Peri	1.00 (0.96–1.05)	1.04* (1.01–1.07)	1.00 (0.99–1.01)	
Delivery by doctor				
Pre	1.00 (Reference)	1.00 (Reference)	1.00 (Reference)	< 0.001
Peri	1.00 (0.97–1.05)	1.05* (1.01–1.10)	1.01 (1.00–1.03)	
Delivery in a public institution				
Pre	1.00 (Reference)	1.00 (Reference)	1.00 (Reference)	0.01
Peri	1.01 (0.95–1.07)	1.02 (0.99–1.05)	0.97*** (0.96–0.99)	
Any PNC				
Pre	1.00 (Reference)	1.00 (Reference)	1.00 (Reference)	0.12
Peri	1.05 (0.99–1.12)	1.03 (0.97–1.10)	0.98 (0.94–1.03)	

^a^ Multilevel modelling estimates adjusted for education, residence, child gender, household wealth status, currently working status and birth order; * < 0.05; ** < 0.01; *** < 0.001; sample size is pre-conflict 2569 and peri-conflict 4641;# joint interaction term explaining the effect of conflict on outcome variable between strata

### Association between conflict and maternal services usage by maternal employment

In Table 5, the interaction test showed that the effect of conflict differed by maternal employment status for SBA (*p* = 0.01) and physician-assisted deliveries (*p* < 0.001). Stratified analysis showed that the increase in the odds of SBA during conflict was relatively greater for employed mothers (OR 1.04; 95% CI 1.01–1.07) than for unemployed mothers (OR 1.01; 95% CI 1.00–1.02) and the increase in the odds of physician-assisted delivery was also greater for employed mothers (OR 1.07; 95% CI 1.02–1.11) than for unemployed mothers (OR 1.01; 95% CI 1.00–1.02).

**Table 5:** Multilevel modelling estimates of the association between conflict and maternal care usage by maternal employment status

Period	Unemployed (*n* = 4640)	Employed (*n* = 802)	Wald *p* value^#^
	OR^a^ (95% CI)	OR^a^ (95% CI)	
4+ ANC visits			
Pre	1.00 (Reference)	1.00 (Reference)	0.99
Peri	1.01 (0.99–1.02)	1.01 (0.98–1.04)	
ANC received from a government provider			
Pre	1.00 (Reference)	1.00 (Reference)	0.92
Peri	0.98 (0.96–1.00)	1.00 (0.93–1.08)	
ANC received from a doctor			
Pre	1.00 (Reference)	1.00 (Reference)	0.08
Peri	1.00 (0.96–1.04)	1.03 (0.98–1.08)	
Delivery by skilled provider			
Pre	1.00 (Reference)	1.00 (Reference)	0.01
Peri	1.01 (1.00–1.02)	1.04* (1.01–1.07)	
Delivery by doctor			
Pre	1.00 (Reference)	1.00 (Reference)	< 0.001
Peri	1.01* (1.00–1.02)	1.07*** (1.02–1.11)	
Delivery in a public institution			
Pre	1.00 (Reference)	1.00 (Reference)	0.20
Peri	0.99 (0.97–1.01)	0.98 (0.91–1.05)	
Any PNC			
Pre	1.00 (Reference)	1.00 (Reference)	0.67
Peri	1.00 (0.98–1.03)	1.04 (0.97–1.11)	

^a^ Multilevel modelling estimates adjusted for education, residence, child gender, household wealth status, currently working status and birth order; * < 0.05; ** < 0.01; *** < 0.001; sample size is pre-conflict 2569 and peri-conflict 4641;# joint interaction term explaining the effect of conflict on outcome variable between strata

### Association between conflict and maternal services usage by household wealth

In Table 6, the joint interaction test showed that the effect of conflict differed by household wealth status for receiving 4+ ANC visits (*p* < 0.001), ANC from a government provider (*p* = 0.004), SBA (*p* < 0.001), and physician-assisted delivery (*p* < 0.001). Stratified analysis showed that the increase in the odds of having any ANC (i.e. either 4+ ANC visits, ANC from government provider, or ANC from a doctor) during conflict was relatively lowest for women from poorest households (OR 1.00; 95% CI 0.98–1.02) than women in all the other wealth quintiles (all with OR 1.01); the reduction in odds of receiving ANC from a government provider during conflict was relatively greater for women from richest households (OR 0.97; 95% CI 0.95–0.98) than women in all other wealth quintiles (all OR 0.99 or greater); the increase in the odds of SBA during conflict was relatively greater for women from less poor households (OR 1.03; 95% CI 1.02–1.05) than women in all other wealth quintiles (all OR 1.02 or less); and the increase in the odds of physician-assisted delivery during conflict was relatively greater for women from less poor (OR 1.03; 95% CI 1.01–1.05) and middle households (OR 1.03; 95% CI 1.01–1.05) than women in other wealth quintiles (all OR 1.02 or less).

**Table 6:** Multilevel modelling estimates of the association between conflict and maternal care usage by household wealth quintile

Period	Poorest (*n* = 1032)	Less poor (*n* = 1079)	Middle (*n* = 1084)	Richer (*n* = 1100)	Richest (*n* = 1147)	Wald *p* value^#^
	OR^a^ (95%CI)	OR^a^ (95%CI)	OR^a^ (95%CI)	OR^a^ (95%CI)	OR^a^ (95%CI)	
4+ ANC visits						
Pre	1.00 (Reference)	1.00 (Reference)	1.00 (Reference)	1.00 (Reference)	1.00 (Reference)	< 0.001
Peri	1.00 (0.98–1.02)	1.01***(1.01–1.02)	1.01 (0.98–1.04)	1.01 (0.99–1.04)	1.01 (0.99–1.02)	
ANC from a government provider						
Pre	1.00 (Reference)	1.00 (Reference)	1.00 (Reference)	1.00(Reference)	1.00 (Reference)	0.004
Peri	0.99 (0.94–1.03)	1.00 (0.98–1.03)	1.01 (0.94–1.08)	0.99 (0.94–1.03)	0.97***(0.95–0.98)	
ANC from a doctor						
Pre	1.00 (Reference)	1.00 (Reference)	1.00 (Reference)	1.00 (Reference)	1.00 (Reference)	0.12
Peri	1.02 (0.95–1.10)	0.95 (0.84–1.06)	1.01 (0.98–1.04)	1.02 (0.98–1.07)	1.01 (0.99–1.03)	
Delivery by skilled provider						
Pre	1.00 (Reference)	1.00 (Reference)	1.00 (Reference)	1.00(Reference)	1.00 (Reference)	< 0.001
Peri	1.01 (0.99–1.04)	1.03***(1.02–1.05)	1.02***(1.01–1.04)	1.00 (0.98–1.02	1.00 (0.99–1.02)	
Delivery by doctor						
Pre	1.00 (Reference)	1.00 (Reference)	1.00 (Reference)	1.00 (Reference)	1.00 (Reference)	< 0.001
Peri	1.01(0.98–1.03)	1.03***(1.01–1.05)	1.03***(1.01–1.05)	1.02 (0.99–1.07)	1.02* (1.00–1.03)	
Delivery in public institution						
Pre	1.00 (Reference)	1.00 (Reference)	1.00 (Reference)	1.00 (Reference)	1.00 (Reference)	0.28
Peri	0.99(0.94–1.05)	1.00 (0.96–1.05)	0.98 (0.93–1.03)	1.04 (0.97–1.10)	0.96 (0.90–1.01)	
Any PNC						
Pre	1.00 (Reference)	1.00 (Reference)	1.00 (Reference)	1.00 (Reference)	1.00 (Reference)	0.08
Peri	1.04 (1.00–1.08)	1.00 (0.98–1.03)	0.99 (0.93–1.06)	1.01 (0.97–1.05)	0.99 (0.93–1.05)	

^a^ Multilevel modelling estimates adjusted for education, residence, child gender, household wealth status, currently working status and birth order; * < 0.05; ** < 0.01; *** < 0.001; sample size is pre-conflict 2569 and peri-conflict 4641;# joint interaction term explaining the effect of conflict on outcome variable between strata

## Discussion

The association between the acute conflict and equity in maternal services usage generally appeared equitable, challenging prevailing assumptions in the literature [42]. A comparison of study findings with those from the 2014 EDHS report, which examined maternal care use over a longer period (2009–2014), helps interpretation of study associations within broader socioeconomic trends. Patterns of equity in maternal care usage during the conflict differed from recent trends in Egypt, indicating vertical equity did not worsen and in some cases improved for specific vulnerable groups during conflict. For example, while associations between conflict and maternal care among socioeconomically advantaged women were minimal, known vulnerable groups (e.g. rural women) had a higher odds of getting maternal care in conflict than pre-conflict. Thus, this study found a relative improvement in vertical equity, as socioeconomically disadvantaged groups such as rural and low-income women had a relatively higher chance of accessing maternal care during conflict (relative to pre-conflict) than their better-off counterparts. However, conflict did not noticeably worsen the chance of maternal care access among socioeconomically advantaged women during the conflict compared to the pre-conflict period.

Recent trends in Egypt indicated higher maternal care use among richer women, while evidence elsewhere indicated that maternal care for both rich and poor women can be adversely affected during conflicts [20]. However, compared with pre-conflict, the odds of receiving maternal care during conflict increased for both poorer and richer women. Unlike recent trends in Egypt, the odds of maternal care increased during conflict as maternal age increased. Research from Nepal, Sri Lanka and Yemen indicated a negative association of conflict with age, while Iraq reported lower use of SBA (22%) among women under 25 [14, 43–49]. Also contrary to recent trends in Egypt, and evidence elsewhere, odds of maternal care use during conflict did not increase with increased educational attainment [2, 21]. Against recent trends, rural women had relatively higher odds of using maternal services during conflict, while evidence elsewhere shows conflict could impact both rural and urban women adversely [29, 42]. Reinforcing recent trends in Egypt, and existing evidence from LMICs, employed mothers had increased odds of maternal service use during conflict [17, 21, 42]. However, the odds of physician-assisted delivery during conflict also increased for unemployed mothers.

Age-related findings were comparable with those from the 2014 DHS report. Both reported that women aged 30–34 were more likely to deliver in public institutions [21]. Evidence suggests that public providers are more frequently chosen by older, poorer, and more rural residents in Asia [11], mainly due to trust in provider behaviour, affordability, and availability in rural areas [4, 50]. Another study in Egypt also indicated a higher reliance on public sector childbirth among women above age 30 [51, 52]. Additionally, the fertility rate in Egypt is somewhat higher among older women living in rural areas, where public-sector facilities are more readily available [9, 15, 23].

This study found lower odds of any PNC visits for women aged 25–29 than for older women during conflict compared to pre-conflict, while recent country trends indicated comparatively higher PNC use in this age group. PNC attendance is typically higher among women who deliver in private institutions in LMICs, particularly in Egypt [15, 53]. An assessment of maternal care in conflict-affected settings indicated that women’s usage depended on perceived need and ability to access care without endangering life [5, 54]. Other studies indicated women may have wanted to reach home safely rather than wait for PNC during conflict [1, 55, 56].

In Egypt, urban areas report better physical access to health centres [23, 25] and fewer socioeconomic and cultural barriers than rural areas [9]. Despite this, rural women had higher odds of using maternal services during the conflict (relative to pre-conflict) than urban women, possibly reinforcing media reports that conflict was more severe in urban areas [30]. The existing literature is inconclusive as to how the severity of conflict affects maternal care. Evidence from acute conflict-affected Nepal, Morocco and Afghanistan indicates that it was not the severity of conflict but rather availability of services that determined maternal usage [43, 55, 57]. Conversely, evidence from 19 conflict-affected sub-Saharan African countries and Sri Lanka indicated that maternal care was more adversely affected in urban areas during severe conflict [12, 14].

Contrary to existing literature in LMICs, including the 2014 DHS report, the odds of maternal care use (i.e. ANC, SBA, or public sector delivery) did not decrease among less-educated women compared with pre-conflict [43, 48, 58, 59]. Pooled odds ratios from a systematic review showed that education level was associated with 20% higher odds of SBA usage during conflicts in Asia and the Middle-East [60]. However, literature also indicates that availability of service and social cohesion can be more relevant during conflict than women’s education status [61]. As the Egyptian conflict was less severe, its potential adverse effect on less educated women could have been very limited.

Qualitative evidence from Egypt and similar settings indicated that some socio-cultural barriers to maternal care that are more frequently experienced by less-educated women can be stronger during conflict [20, 48]. For example, the literature indicates that less-educated women in Asia have relatively weak autonomy in decision-making, travel, and purchasing power, especially during conflict [61, 62]. Thus, compared to more-educated women, they tend to use public rather than more expensive private facilities. However, in this study context, this use of public sector among less educated women could be more of a reflection of the recent trends in the country.

Similar to the 2014 DHS report and a study from Yemen, employed women had relatively higher odds than unemployed women of using SBA and physician-assisted delivery during conflict [21, 46]. However, unlike the DHS report, this study did not find significantly greater ANC and PNC use among employed women during conflict compared to pre-conflict. The higher odds of physician-assisted delivery suggested employed women had more access to private institutional care during conflict. Another study from Egypt reported working women were more likely to use maternal care and physician-assisted delivery from private institutions irrespective of conflict [9, 63]. However, a slight increase in the odds of physician-assisted delivery among unemployed women during the conflict compared with pre-conflict is worth noting. Given the perceived complications in delivery care, women may have felt relatively safer using physician services than those of other types of providers during the conflict [42]. Alternatively, the extensive policy attention on quality of maternal care could have prompted them to seek physician services [64]. This finding could be also a reflection of the recent trends in the country, as Egypt in general depends largely on private sector and physicians for maternal care [21].

Unlike the 2014 DHS report and other studies in Egypt, women from poor households had higher odds of using maternal care during conflict (relative to pre-conflict) than women from wealthy households, possibly reinforcing the role of conflict in driving maternal care beyond the level of affordability [11, 20, 21]. This finding supported the literature, which indicates that the effect of household wealth on maternal services use during conflict is unpredictable, due to emergency nature of maternal care and households’ perceived need for care [2]. Pooled odds ratios from a systematic review showed that household wealth was not associated with increased odds of SBA usage during conflicts in Asia and the Middle-East [60]. In Egypt, poor women are more concentrated in rural areas, while conflict was also less severe in rural areas, possibly supporting this higher use among poor women [20]. However, the literature does indicate that women accessing care irrespective of their financial status is regressive, especially in an inequitable health care system [2, 4]. Although poor women used services, given the regressive health financing system and inadequacy of supplies in public hospitals, there could have been a higher chance of financial catastrophe, which was not assessable [1, 3, 19].

### Policy and research implications

Study findings show that existing equity patterns in maternal care changed unpredictably during the conflict. If the healthcare delivery system is well developed with progressive health financing, the scope for a conflict to cause large inequities is limited. However, given the limited availability of quality maternal care, inequities in service delivery, and regressive health financing in Egypt, maternal policy could benefit from specific in-built equity strategies to address unpredictable effects of conflict on equity [2, 4]. For example, strategic involvement of community-based groups, volunteers, and local providers has helped pregnant women during emergencies [3]. Depending on the severity of conflict and women’s relative vulnerability, failure to implement remedial measures could worsen equity [2, 4].

Experiences in several countries affected by acute and sporadic conflicts (e.g. Nepal, Myanmar) showed that post-conflict reconstruction could offer opportunities to build more equitable health systems than existed previously [17, 61]. The commitment shown by Egyptian policy-makers in implementing multi-sectorial policy measures to address health inequities is worth acknowledging [10, 65]. Improved maternal care use among socioeconomically disadvantaged groups could be partially due to this increased policy attention. Increasing the involvement of non-state actors may strengthen the government’s equity-driven initiatives further. For instance, active participation of civil society in policy-making may inspire maternal health policies to be more equity-focused [30]. Given the financial and technical constraints in the public health system, development partners and the private sector could leverage funding and technical capacity to implement equitable maternal care strategies [64]. Enhancing the capacity of providers and community-based networks could reduce access barriers for previously marginalised groups [30].

Due to data constraints, this study did not assess the association between conflict, out-of-pocket expenditure, and financial catastrophes due to maternal care. Egypt’s proportion of out-of-pocket healthcare expenditure is high at more than 70%, while its financial risk-protection measures are still evolving [41]. User fees and lack of pre-payment systems are known limitations in the Egyptian health system [66, 67]. During major conflicts, financial access to care typically deteriorates due to collapsing livelihoods and healthcare delivery services [2]. Though the 2011–2012 Egyptian conflict was not particularly severe, maternal needs could have engendered financial hardship, particularly among poorer groups [27, 68–70].

In-depth research is needed to explore the underlying drivers of maternal care equity during future conflicts [42]. It should be noted that socioeconomic adversity in Egypt is more concentrated in the Rural South Region, which was relatively less conflict-affected than the more affluent urban areas [11, 20]. This could be a reason for maternal care among vulnerable groups not being more significantly adversely affected by conflict in this study. Egypt has recently been implementing several maternal and child health initiatives in the Rural South Region [20], which could have positively influenced maternal care among socioeconomically disadvantaged groups. Additional evidence is needed on the differential association of conflict and quality of maternal care used by different groups. Assessing the equity dimension in quality of maternal care would help understanding of conflict’s potential effect on maternal health status among different groups [71]. The literature indicates that LMICs generally provide relatively low-quality maternal services to economically poorer women, as is reportedly the case in Egypt [70, 72].

### Limitations

Several potential limitations relate to the nature of the data. First, as DHS data were not specifically collected to assess the effects of conflict, customising data led to omitting relevant ANC and PNC variables due to incompatibility with a before-and-after analysis. Second, DHS data were self-reported and described details of maternal care-seeking in previous years, possibly leading to recall or social desirability biases [73]. However, a validation study in LMICs found moderate to high sensitivity and moderate validity for self-reported coverage of maternal care in surveys [74, 75]. DHS data were representative of childbirth experiences in the general population, and DHS employed standardised procedures to ensure data quality and tools were rigorously tested across time. Third, the EDHS wealth index is potentially biased against rural households, by including more items or utilities (e.g. electrical appliances) suited to urban populations [73]. Fourth, underlying temporal trends could have influenced the measurement of effect size, though the period under consideration was too short for a large temporal trend to have occurred [75]. Fifth, as the effect size found was relatively small, qualitative exploration would have been helpful to generate additional explanatory evidence. Sixth, given the country-wide geographical spread of the conflict and lack of data on region-specific exposures, this study considered all women to be equally exposed to conflict and could not differentiate level of exposure. Finally, the number of outcomes and potential effect modifiers considered meant that multiple statistical tests were performed, increasing the likelihood of finding evidence of effect modification by chance alone.

## Conclusions

Despite limitations, this study is a rare attempt to measure the association between an acute conflict and equity of maternal services usage. Maternal care use during conflict was generally vertically equitable in Egypt, as opposed to prevailing evidence in LMICs. Authors call for specific equity strategies in maternal policy to help address the unpredictable effects of conflict on equity of health services provision, such as those for maternal care examined here. Additional evidence is needed on how conflict affects out-of-pocket expenditure, financial catastrophe, and quality of maternal care among women from different socioeconomic groups.

## Abbreviations

ANC: Ante natal care
CI: Confidence interval
DHS: Demographic and health survey
EDHS: Egypt demographic and health survey
OR: Odds ratio
PNC: Post natal care
SBA: Skilled birth attendance
SDG: Sustainable development goals
UN: United Nations
